# Antiproliferation for Breast Cancer Cells by Ethyl Acetate Extract of *Nepenthes thorellii* x (*ventricosa* x *maxima*)

**DOI:** 10.3390/ijms20133238

**Published:** 2019-07-01

**Authors:** Fu Ou-Yang, I-Hsuan Tsai, Jen-Yang Tang, Ching-Yu Yen, Yuan-Bin Cheng, Ammad Ahmad Farooqi, Shu-Rong Chen, Szu-Yin Yu, Jun-Kai Kao, Hsueh-Wei Chang

**Affiliations:** 1 Division of Breast Surgery and Department of Surgery, Kaohsiung Medical University Hospital, Kaohsiung 80708, Taiwan; 2 Cancer Center, Kaohsiung Medical University Hospital, Kaohsiung 80708, Taiwan; 3 Department of Biomedical Science and Environmental Biology, Kaohsiung Medical University, Kaohsiung 80708, Taiwan; 4 Department of Radiation Oncology, Faculty of Medicine, College of Medicine, Kaohsiung Medical University, Kaohsiung 80708, Taiwan; 5 Department of Radiation Oncology, Kaohsiung Medical University Hospital, Kaohsiung 80708, Taiwan; 6 Department of Oral and Maxillofacial Surgery Chi-Mei Medical Center, Tainan 71004, Taiwan; 7 School of Dentistry, Taipei Medical University, Taipei 11050, Taiwan; 8 Graduate Institute of Natural Products, Kaohsiung Medical University, Kaohsiung 80708, Taiwan; 9 Institute of Biomedical and Genetic Engineering (IBGE), Islamabad 44000, Pakistan; 10 Institute of Biomedical Sciences, National Chung Hsing University, Taichung 40227, Taiwan; 11 Pediatric Department, Children’s Hospital, Changhua Christian Hospital, Changhua 50006, Taiwan; 12 School of Medicine, Kaohsiung Medical University, Kaohsiung 80708, Taiwan; 13 Drug Development and Value Creation Research Center, Kaohsiung Medical University, Kaohsiung 80708, Taiwan; 14 Institute of Medical Science and Technology, National Sun Yat-sen University, Kaohsiung 80424, Taiwan; 15 Department of Medical Research, Kaohsiung Medical University Hospital, Kaohsiung 80708, Taiwan

**Keywords:** breast cancer, oxidative stress, carnivorous plants, natural product

## Abstract

Extracts from the Nepenthes plant have anti-microorganism and anti-inflammation effects. However, the anticancer effect of the Nepenthes plant is rarely reported, especially for breast cancer cells. Here, we evaluate the antitumor effects of the ethyl acetate extract of *Nepenthes* *thorellii* x (*ventricosa* x *maxima*) (EANT) against breast cancer cells. Cell viability and flow cytometric analyses were used to analyze apoptosis, oxidative stress, and DNA damage. EANT exhibits a higher antiproliferation ability to two breast cancer cell lines (MCF7 and SKBR3) as compared to normal breast cells (M10). A mechanistic study demonstrates that EANT induces apoptosis in breast cancer cells with evidence of subG1 accumulation and annexin V increment. EANT also induces glutathione (GSH) depletion, resulting in dramatic accumulations of reactive oxygen species (ROS) and mitochondrial superoxide (MitoSOX), as well as the depletion of mitochondrial membrane potential (MMP). These oxidative stresses attack DNA, respectively leading to DNA double strand breaks and oxidative DNA damage in γH2AX and 8-oxo-2′deoxyguanosine (8-oxodG) assays. Overall these findings clearly revealed that EANT induced changes were suppressed by the ROS inhibitor. In conclusion, our results have shown that the ROS-modulating natural product (EANT) has antiproliferation activity against breast cancer cells through apoptosis, oxidative stress, and DNA damage.

## 1. Introduction

Breast cancer is the most common cancer in women, comprises 30% of all new female cancer cases, and accounts for 15% of all female cancer-related deaths [1]. Breast cancer is complex and an effective cure remains elusive. Resistance to clinical drugs and radiation reduces the therapeutic effect against breast cancer and is partly attributed to the loss of apoptosis function [2]. Accordingly, apoptosis-inducing drugs may improve the therapeutic effect against breast cancer cells.

Accumulating evidence suggests that natural products contain many bioactive components for cancer prevention and therapy, and sometimes provide advantages over isolated compounds. This is partly because natural products consist of many bioactive components that can suppress the function of multiple targets [3]. Natural products provide valuable resources for drug discovery for breast cancer treatment through apoptosis [2,4,5,6,7]. For example, *Phyla nodiflora* L. extracts were found to inhibit cell proliferation by inducing apoptosis in human breast cancer cells [2]. The study of additional natural products for drug discovery against breast cancer cells is thus warranted.

*Nepenthes* (also known as tropical pitcher plants) are tropical carnivorous plants. *Nepenthes* comprise a number of natural and cultivated hybrids and present diverse species development. Many species of *Nepenthes* are commonly used in herbal medicine in several Southeast Asian countries [8]. Some types of extracts from *Nepenthes* are known to have anti-bacterial and anti-fungal properties. For example, methanolic extract of *N. bicalcarata* inhibited growth of gram-positive bacteria (*Staphylococcus aureus*, *Bacillus subtilis* and *B. spizizenii*) with the minimum inhibitory concentration (MIC) at 256 μg/mL [9]. Hexane extract of *N. ventricosa* x *maxima* inhibited the growth of several species of fungi such as *Alternaria alternata*, *Aspergillus niger*, *Bipolaris oryzae*, *Fusarium oxysporum*, *Phytophthora capsici*, *Rhizoctonia solani*, *Rhizopus stolonifer* var. stolonifera, and *Sclerotinia sclerotiorum* with MIC values ranging from 7.2 to 43.7 μg/mL [10]. *Nepenthes* extracts have been reported to suppress inflammation [11]. Anti-inflammation drugs frequently show anticancer effects [12,13,14]. Recently, the methanol extract and its sequential partitions of *N. alata* Blanco as well as its bioactive compound plumbagin demonstrated the anti-breast-cancer effect [15]. Therefore, we hypothesize that extracts from other *Nepenthes hybrid* may have an anticancer effect against breast cancer cells.

This study evaluates the antiproliferation effect from an ethyl acetate extract of *Nepenthes thorellii x (ventricosa x maxima)* (EANT) on breast cancer cells. The underlying mechanisms of antiproliferation (e.g., cell viability, apoptosis, oxidative stress, and DNA damage) were determined on breast cancer cells following EANT treatment.

## 2. Results

### 2.1. The Identified Components from Fingerprint Profiles of EANT

According to HPLC fingerprinting assay (Supplementary Figure S1), the major bioactive components of EANT are isoplumbagin, *cis*-isoshinanolone, quercetin 3-*O*-(6″-*n*-butyl β-D-glucuronide), and fatty acids. In the present study, we focused on the antiproliferation effect of breast cancer cells using EANT and therefore the detailed involvement of these bioactive components on anti-breast cancer effect was not addressed.

### 2.2. EANT Preferentially Inhibits Viability of Breast Cancer Cells

**Figure 1:**
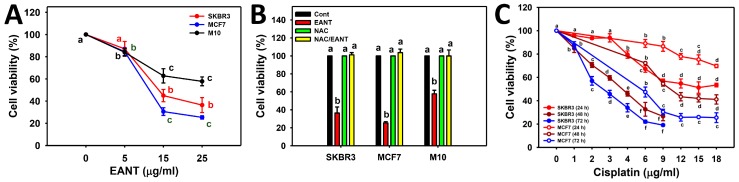
Cell viability following ethyl acetate extract of *Nepenthes thorellii x (ventricosa x maxima)* (EANT) treatment. (**A**) Cell viability of breast cancer cells (MCF7 and SKBR3) and breast normal cells (M10) treated with 0 (control with DMSO only), 5, 15, and 25 μg/mL of EANT for 24 h. (**B**) Cell viability of breast cancer cells after NAC pretreatment (2 mM for 1 h) and EANT post-treatment (25 μg/mL for 24 h), i.e., NAC/EANT. (**C**) Cell viability of breast cancer cells treated with different concentrations of cisplatin for 24, 48, and 72 h. For each cell line, treatments labeled without the same lower-case letters indicate significant difference. *p* < 0.05~0.0001. Data, mean ± SD (*n* = 3).

In Figure 1A, EANT dose-dependently more significantly reduces the viability (%) of two breast cancer cells (MCF7 and SKBR3) than normal breast cells (M10). In Figure 1B, the viability reducing effects of EANT are suppressed by the inhibitor pretreatments for ROS (NAC). Accordingly, the role of oxidative stress and apoptosis in EANT-treated breast cancer cells warrants further detailed investigation. For comparison to clinical drugs, the cell viability of breast cancer cells treated with cisplatin was performed (Figure 1C). The drug sensitivity of EANT in breast cancer cells is higher than that of cisplatin at 24 h treatment. The drug sensitivity of cisplatin in breast cancer cells at 48 and 72 h is higher than that of 24 h treatment.

### 2.3. EANT Changes Cell Cycle Distribution in Breast Cancer Cells

**Figure 2:**
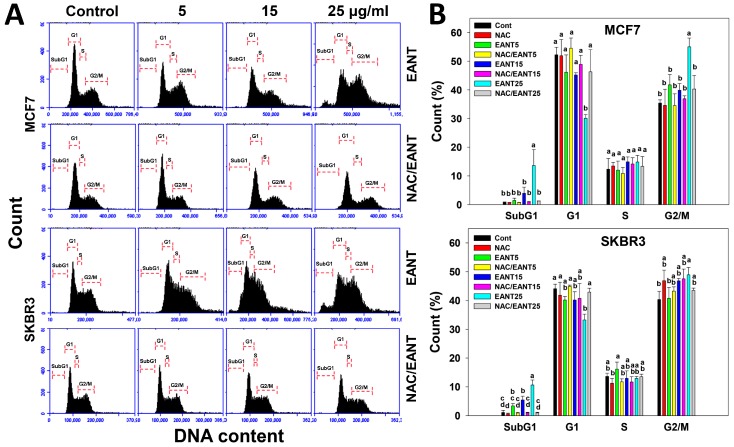
Cell cycle change after EANT treatment. (**A**,**B**) Cell cycle distribution patterns and statistics. Without or with NAC pretreatment, breast cancer cells (MCF7 and SKBR3) were treated with 0 (control with DMSO only), 5, 15, and 25 μg/mL of EANT for 24 h, i.e., EANT vs. NAC/EANT. For each cycle phase, treatments labeled without the same lower-case letters indicate significant difference. *p* < 0.05~0.0001. Data, mean ± SD (*n* = 3). Positive controls for subG1 accumulation and G2/M arrest were provided in the Supplementary Figure S2A,B.

Figure 2A shows the flow cytometry patterns of cell cycle distribution in breast cancer cells (MCF7 and SKBR3) without (up) or with (down) NAC pretreatment. In Figure 2B, the subG1 and G2/M population gradually accumulates and the G1 population gradually decreases in breast cancer cells after EANT treatments. After NAC pretreatments, the subG1 accumulation and cell cycle disturbance recover to the normal distribution as control.

### 2.4. EANT Induces Apoptosis in Breast Cancer Cells

**Figure 3:**
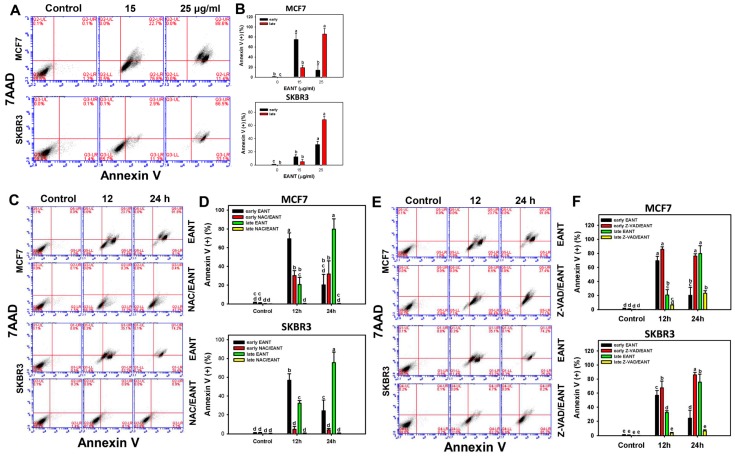
Apoptosis change of annexin V/7AAD after EANT treatment. (**A**,**B**) Concentration effect of EANT on Annexin V/7AAD patterns and statistics. Breast cancer cells (MCF7 and SKBR3) were treated with control with DMSO only and EANT (15 and 25 μg/mL) for 24 h. Annexin V (+)/7AAD (−) and annexin V (+)/7AAD (+) were respectively regarded as early and later apoptosis. (**C**–**F**) Time course effect of EANT on Annexin V/7AAD patterns and statistics. Without or with (**C**,**D**) NAC pretreatment or (**E**,**F**) Z-VAD pretreatment, breast cancer cells (MCF7 and SKBR3) were treated with 25 μg/mL of EANT for 0 (control with DMSO only), 12, and 24 h. For each cell line, treatments labeled without the same lower-case letters indicate significant difference. *p* < 0.01~0.0001. Data, mean ± SD (*n* = 3). Positive controls for apoptosis are provided in Supplementary Figure S2C,D.

The possibility that subG1 accumulation may lead to apoptosis was further examined by flow cytometry. Figure 3A shows the flow cytometry patterns of annexin V/7AAD in breast cancer cells (MCF7 and SKBR3). In Figure 3B (top part), the early apoptosis (%) (annexin V (+)/7AAD (-)) of MCF7 cells is dramatically increased to about 80% in 15 μg/mL of EANT and its late apoptosis (%) (annexin V (+)/7AAD (+)) is increased to 20% compared to the control. In Figure 3B (bottom part), the early and late apoptosis (%) of SKBR3 cells is only mildly increased in 15 μg/mL of EANT compared to the control. In a higher concentration (25 μg/mL), EANT is more likely to induce late apoptosis than early apoptosis in both breast cancer cells.

Figure 3C,E illustrate the roles of ROS and apoptosis effects on flow cytometry patterns of annexin V/7AAD in breast cancer cells (MCF7 and SKBR3). At 12 h of EANT treatment, the early apoptosis population exceeds the late apoptosis population in breast cancer cells. At 24 h of EANT treatment, the early apoptosis population shifts to late apoptosis in breast cancer cells. In Figure 3D,F, the early apoptosis population, that is, annexin V(+)/7AAD (−) (%), increases dramatically for 12 h and then declines at 24 h in breast cancer cells. The late apoptosis population, that is, annexin V(+)/7AAD (+) (%), increases dramatically for 12 and 24 h in breast cancer cells. 

For EANT post-treatment for 12 h, NAC or Z-VAD pretreatment inhibits both early and late apoptosis populations. For EANT post-treatment for 24 h, NAC or Z-VAD pretreatment inhibits late apoptosis populations and the late apoptosis population shifts to early apoptosis.

### 2.5. EANT Induces ROS Production and GSH Depletion in Breast Cancer Cells

**Figure 4:**
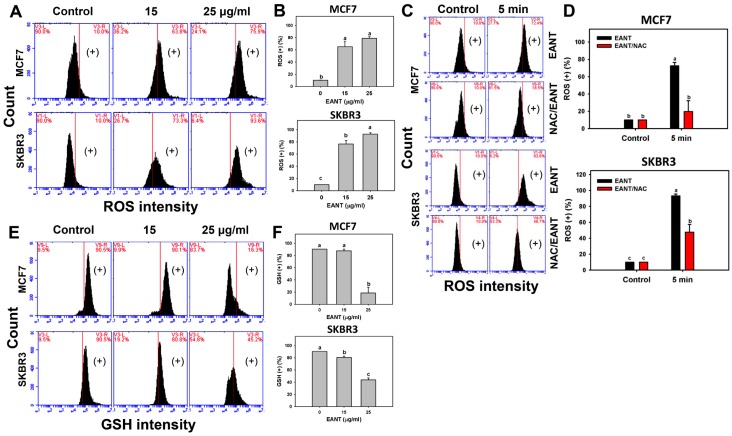
Reactive oxygen species (ROS) and glutathione (GSH) changes after EANT treatment. (**A**,**B**) Concentration effect of EANT on ROS patterns and statistics. Breast cancer cells (MCF7 and SKBR3) were treated with control with DMSO only and EANT (15 and 25 μg/mL) for 5 min. (**C**,**D**) Time course effect of EANT on ROS patterns and statistics. Without or with NAC pretreatment, breast cancer cells (MCF7 and SKBR3) were treated with control with DMSO only and 25 μg/mL of EANT for 5 min. (**E**,**F**) The concentration effect of EANT on GSH patterns and statistics. Breast cancer cells were treated with control with DMSO only and EANT for 24 h. For each cell line, treatments labeled without the same lower-case letters indicate significant difference. *p* < 0.05~0.0001. Data, mean ± SD (*n* = 3). (+) indicates the ROS or GSH (+) population. Positive controls for ROS and GSH were provided in Supplementary Figure S2E–H.

Since NAC reverts the EANT induced changes of cell viability, cell cycle distribution, and apoptosis, the status of oxidative stress such as the ROS level in EANT-treated breast cancer cells warrants detailed investigation. Figure 4A,C respectively show flow cytometry patterns of ROS in breast cancer cells (MCF7 and SKBR3) without or with NAC pretreatment. In Figure 4B, the ROS (+) (%) is dramatically increased in both 15 and 25 μg/mL of EANT. In Figure 3D, the ROS (+) (%) increases dramatically within 5 min and is suppressed by NAC pretreatment in breast cancer cells (MCF7 and SKBR3).

Because glutathione (GSH) has a ROS-scavenging function [16], the status of GSH level in EANT-treated breast cancer cells warrants detailed investigation. Figure 4E shows flow cytometry patterns of GSH in breast cancer cells (MCF7 and SKBR3). In Figure 4F, the GSH (+) (%) is dramatically decreased in 25 μg/mL of EANT in breast cancer cells.

### 2.6. EANT Induces MitoSOX Production and MMP Reduction in Breast Cancer Cells

**Figure 5:**
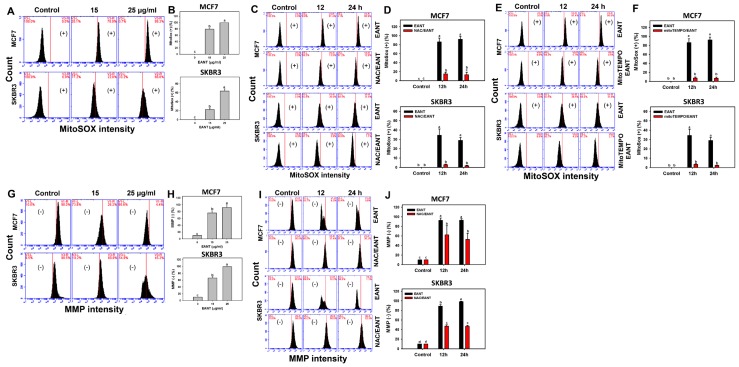
MitoSOX and MMP change after EANT treatment. (**A**,**B**) The concentration effect of EANT on MitoSOX patterns and statistics. Breast cancer cells (MCF7 and SKBR3 were treated with 0 (control with DMSO only), 15, and 25 μg/mL of EANT for 24 h. (**C**–**F**) Time course effect of EANT on MitoSOX patterns and statistics. Without or with (**C** and **D**) NAC pretreatment or (**E**,**F**) MitoTEMPO pretreatment, breast cancer cells (MCF7 and SKBR3) were treated with 25 μg/mL of EANT for 0 (control with DMSO only), 12, and 24 h. Annexin V (+) was counted to apoptosis. (**G**,**H**) The concentration effect of EANT on MMP patterns and statistics. Breast cancer cells (MCF7 and SKBR3) were treated with EANT for 24 h. (**I**,**J**) Time course effect of EANT on MMP patterns and statistics. Without or with NAC pretreatment, breast cancer cells (MCF7 and SKBR3) were treated with 25 μg/mL of EANT for 0 (control with DMSO only), 12, and 24 h. For each cell line, treatments labeled without the same lower-case letters indicate significant difference. *p* < 0.05~0.0001. Data, mean ± SD (*n* = 3). (+) or (−) respectively indicates the MitoSOX (+) or MMP (−) population. Positive controls for MitoSOX and MMP were provided in Supplementary Figure S2I–L.

Since NAC reverts the EANT induced changes to cell viability, cell cycle distribution, and apoptosis, the status of oxidative stress such as MitoSOX and MMP levels in EANT-treated breast cancer cells warrants detailed investigation. Figure 5A shows flow cytometry patterns of MitoSOX in breast cancer cells (MCF7 and SKBR3). In Figure 5B, the MitoSOX (+) (%) is dramatically increased in both 15 and 25 μg/mL of EANT. Figure 5C,E respectively show flow cytometry patterns of MitoSOX in breast cancer cells (MCF7 and SKBR3) without or with NAC or MitoTEMPO. In Figure 5D,F, the MitoSOX (+) (%) of breast cancer cells are suppressed by the inhibitor pretreatments for ROS (NAC) or MitoSOX (MitoTEMPO).

Figure 5G shows flow cytometry patterns of MMP in breast cancer cells (MCF7 and SKBR3). In Figure 5H, the MMP (−) (%) is dramatically increased in both 15 and 25 μg/mL of EANT. Figure 5I shows the time course for flow cytometry patterns of MMP in breast cancer cells (MCF7 and SKBR3) without or with NAC. In Figure 5J, the MMP (−) (%) of breast cancer cells is increased at 12 and 24 h and is suppressed by the NAC pretreatment.

### 2.7. EANT Induces DNA Damage in Breast Cancer Cells

**Figure 6:**
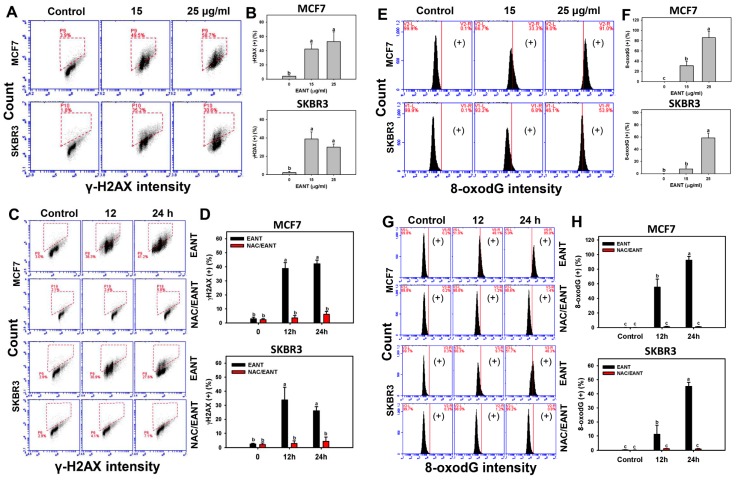
γH2AX and 8-oxodG changes after EANT treatment. (**A**,**B**) The concentration effect of EANT on γH2AX patterns and statistics. Breast cancer cells (MCF7 and SKBR3) were treated with 0 (control with DMSO only), 15, and 25 μg/mL of EANT for 24 h. The dashed box indicates the γH2AX (+) population. (**C**,**D**) Time course effect of EANT on γH2AX patterns and statistics. Without or with NAC pretreatment, breast cancer cells (MCF7 and SKBR3) were treated with 25 μg/mL of EANT for 0 (control with DMSO only), 12 and 24 h. (**E**,**F**) The concentration effect of EANT on 8-oxodG patterns and statistics. Breast cancer cells (MCF7 and SKBR3) were treated with control with DMSO only and EANT for 24 h. (**G**,**H**) Time course effect of EANT on 8-oxodG patterns and statistics. Without or with NAC pretreatment, breast cancer cells (MCF7 and SKBR3) were treated with 25 μg/mL of EANT for 0 (control with DMSO only), 12, and 24 h. For each cell line, treatments labeled without the same lower-case letters indicate significant difference. *p* < 0.01~0.001. Data, mean ± SD (n = 3). (+) indicates the 8-oxodG (+) population. Positive controls for γH2AX and 8-oxodG were provided in Supplementary Figure S2M–P.

ROS is a DNA damage factor [17] and the DNA damage status in EANT-treated breast cancer cells warrants detailed investigation. Figure 6A shows flow cytometry patterns of γH2AX in breast cancer cells (MCF7 and SKBR3). In Figure 6B, the γH2AX (+) (%) is dramatically increased in both 15 and 25 μg/mL of EANT. Figure 6C shows time course for flow cytometry patterns of γH2AX in breast cancer cells (MCF7 and SKBR3) without or with NAC pretreatment. In Figure 6D, the γH2AX (+) (%) of breast cancer cells are suppressed by NAC pretreatment.

Figure 6E shows flow cytometry patterns of 8-oxodG in breast cancer cells (MCF7 and SKBR3). In Figure 6F, the 8-oxodG (+) (%) is gradually increased in both 15 and 25 μg/mL of EANT. Figure 6G shows time course for flow cytometry patterns of 8-oxodG in breast cancer cells (MCF7 and SKBR3) without or with NAC pretreatment. In Figure 6H, the 8-oxodG (+) (%) of breast cancer cells is increased at 12 and 24 h and is suppressed by NAC pretreatment.

## 3. Discussion

### 3.1. EANT Preferentially Inhibits Proliferation of Breast Cancer Cells

The antiproliferation effect of cancer cells using Nepenthes has been rarely reported. The current study demonstrates that EANT inhibited the cell proliferation of breast cancer cells more than that of normal breast cells at higher concentrations (15 and 25 μg/mL) (Figure 1A). IC_50_ values of EANT are respectively 10 and 15 μg/mL for MCF7 and SKBR3 cells at 24 h MTS assay. For comparison to extracts from other Nepenthes such as *N. alata* Blanco, IC_50_ values at 24 h MTT assay using MCF7 cells showed 99.78 μg/mL for its methanol extract and showed 36.60, 90.02, 61.13, and > 100 μg/mL for its CH_2_CL_2_, EtOAc, n-BuOH, and H_2_O fractions, respectively [15]. These results suggest that our developed ethyl acetate extract of *N. thorellii* x (*ventricosa* x *maxima*), that is, EANT, has higher sensitivity to breast cancer MCF7 cells than that of *N. alata* Blanco. It is possible that extracts from different Nepenthes may have different components or proportions leading to different drug sensitivity to breast cancer cells.

Antimalarial naphthoquinones such as plumbagin, 2-methylnaphthazarin, octadecyl caffeate, isoshinanolone, and droserone were isolated from *N. thorelii* [18]. Several Nepenthes plants reported that plumbagin is one of the main bioactive components [19,20]. Plumbagin showed IC_50_ values of 1.155 and 0.658 μg/mL to breast cancer MCF7 cells and noncancerous MCF-10A cells was 2.548 and 1.882 μg/mL at 24 and 48 h treatments, respectively [15]. In the current study, we found that the major bioactive components of EANT identified by HPLC fingerprinting method were isoplumbagin [21], *cis*-isoshinanolone [22], and quercetin 3-*O*-(6″-*n*-butyl β-D-glucuronide) [23] (Supplementary Figure S1). Isoplumbagin also isolated from the common leadwort (*Plumbago europaea*) and showed an anti-Candida effect [21], however, its anticancer effect was rarely reported. *cis*-isoshinanolone also isolated from the plant (*Diospyros shimbaensis*) and showed antioxidant property without testing anticancer effect [22]. Quercetin 3-*O*-(6″-*n*-butyl β-D-glucuronide) also isolated from a slow-growing small leafless desert shrub (*Calligonum polygonoides*) and showed cytotoxic effect against liver cancer HepG2 and breast cancer MCF7 cells [23], that is, 60.46 and 61.4 μg/mL at 72 h sulphorhodamine-B assay. It warrants the detailed investigation for the cytotoxic effects of these components in EANT to breast cancer cells in future.

For comparison to clinical drugs, the IC_50_ value of cisplatin is 15 μg/mL for SKBR3 cells but it is undetectable for MCF7 cells (Figure 1C). IC_50_ value of cisplatin at 48 and 72 h MTS assay is 3.71 and 2.69 μg/mL for SKBR3 cells and 10.45 and 6 μg/mL for MCF7 cells (Figure 1C). Similarly, IC_50_ values of cisplatin at 72 h treatment by MTT assay are 3.36 and 4.44 μg/mL [24] for SKBR3 and MCF7 cells. Although the IC_50_ values of EANT at 24 h treatment are higher than that of cisplatin at 72 h treatment, their treatment times are different. For 24 h treatment, EANT has higher drug sensitivity to MCF7 cells and shows similar sensitivity to SKBR3 cells compared to cisplatin.

### 3.2. EANT Induces Oxidative Stress on Breast Cancer Cells

Oxidative stress is an imbalance status between ROS production and antioxidant response [25]. When ROS is overproduced, the cellular antioxidant system is unable to remove extra ROS to steady its state, thus reducing the antioxidant level. The current study found that intracellular ROS and mitochondrial superoxide MitoSOX were upregulated and MMP was downregulated following EANT treatment. These results suggest that EANT induces oxidative stress in breast cancer cells (MCF7 and SKBR3). These oxidative stress changes were validated by NAC or MitoTEMPO pretreatments.

GSH is an ROS scavenging antioxidant in cells [16] to maintain oxidative homeostasis. When the ROS level exceeds the ROS cleanness ability of GSH, the intracellular ROS level may increase and the GSH level may decrease. Natural products have been reported to inhibit cellular antioxidant system of cancer cells and lead to cell death for cancer treatments [26]. For example, ethanolic extract of red algae *Gracilaria tenuistipitata* can increase ROS production and decrease the GSH level of oral cancer cells [27]. Consistently, we found that GSH level declined dramatically at higher concentration of EANT (25 μg/mL). It is noted that the GSH level was not completely depleted in breast cancer cells (MCF7 and SKBR3) following EANT treatment. In addition to GSH, this warrants a detailed investigation for the involvement of other non-GSH members in antioxidant systems.

Plumbagin is a common naphthoquinone in Nepenthes, however, isoplumbagin but not plumbagin is identified in EANT. Plumbagin was reported to bind to GSH [28], behave as electrophile against GSH [29], and induce GSH depletion of leukemia Kasumi-1 [30] and U937 [31] cells. However, the GSH effect of isoplumbagin remains unclear. Accordingly, the role of the naphthoquinones isoplumbagin in cytotoxic effects against breast cancer cells needs detailed investigation in future. Moreover, the possible involvement of the other main compounds (*cis*-isoshinanolone and quercetin 3-*O*-(6″-*n*-butyl β-D-glucuronide)) in EANT-induced cytotoxicity of breast cancer cells cannot be excluded.

### 3.3. EANT Induces Oxidative Stress-Mediated Apoptosis and DNA Damage on Breast Cancer Cells

Oxidative stress is an activator for triggering apoptosis [32]. SubG1 accumulation is one of the apoptosis indicators [33]. In the current study, EANT partly induces subG1 accumulation and increases the intensity of annexin V. These apoptosis events were suppressed by NAC pretreatment, suggesting that EANT induces apoptosis of breast cancer cells depending on oxidative stress. 

Moreover, oxidative stress is an activator for triggering DNA damage [34]. In the current study, the γH2AX marker [35] for DNA double strand breaks was induced in breast cancer cells after EANT treatment. The role of oxidative stress in DNA double strand breaks was further confirmed by evidence that NAC pretreatment suppresses the γH2AX intensity in breast cancer cells (MCF7 and SKBR3) following EANT treatment. This finding raises the possibility that oxidative stress may attach DNA to generate other types of DNA damage. In the example of 8-oxodG [36], we further demonstrated that oxidative DNA damage is also induced in breast cancer cells after EANT treatment. Again, this type of oxidative DNA damage in breast cancer cells is suppressed by NAC pretreatment. Therefore, EANT induces apoptosis and DNA damage to breast cancer cells through oxidative stress.

### 3.4. Conclusion

Extracts from *Nepenthes* plants have been reported to have anti-microorganism effects, but the anticancer effect of *Nepenthes* has rarely been investigated. This study examines the anticancer effect of *Nepenthes* on breast cancer cells in the example of ethyl acetate fraction of *N. thorellii* x *(ventricosa* x *maxima)* (EANT). EANT shows a greater antiproliferation effect against breast cancer cells than for normal breast cells. Different flow cytometric assays show that EANT induces oxidative stress and apoptosis in breast cancer cells and these changes were suppressed by their inhibitors. In conclusion, EANT exerts an antiproliferation effect on breast cancer cells and the detailed mechanisms as mentioned are explored in a ROS-dependent manner.

## 4. Materials and Methods

### 4.1. Nepenthes Extraction and Inhibitors

The aerial parts of *Nepenthes thorellii* x *(ventricosa* x *maxima)* were collected from Dr. Celica Koo Botanic Conservation Center (KBCC), Pingtung County, Taiwan, in October 2014. This *Nepenthes* hybrid plant was identified by KBCC researcher Ray-Hsuan Kuo, marked with the voucher number K47512 EA, and stored in the Graduate Institute of Natural Products, Kaohsiung Medical University.

The aerial part of plant material (500 g) was sliced and immersed in MeOH (1 L) for three days. The organic solvent was removed by a rotavapor (Heidolph, Schwabach, Germany) to afford a methanol extract (25 g). This extract was partitioned between water and ethyl acetate (EA). The EA-soluble fraction of *N. thorellii* x *(ventricosa* x *maxima)* (9.5 g) is referred to here as EANT. EANT was stored in −20 °C and dissolved in dimethyl sulfoxide (DMSO) prior to experimentation. All treatments have the same concentration of DMSO, that is, 0.05%.

To examine the role of oxidative stress, cells were pretreated with 2 mM ROS inhibitor *N*-acetylcysteine (NAC) (Sigma-Aldrich; St Louis, MO, USA) [37] for 1 h. The role of mitochondrial oxidative stress was also examined by pretreating cells with 10 μM of the mitochondrial superoxide inhibitor MitoTEMPO (Cayman Chemical, Ann Arbor, Michigan, USA) [38] for 1 h. The role of apoptosis was examined by pretreating cells with 20 μM of an apoptosis inhibitor Z-VAD-FMK (Z-VAD) (Selleckchem. com; Houston, TX, USA) [39] for 2 h.

### 4.2. HPLC Fingerprint Profile of EANT

In order to understand the abundance and distribution of the components of EANT, the fingerprint profile of EANT was studied. A Shimadzu LC-20AD prominence liquid chromatograph HPLC equipped with SPD-M10A VP diode array detector was used for investigation. Injection of EANT (volume = 100 μL, concentration = 1 mg/mL, in MeOH) was performed by a Shimadzu SIL-10AD VP auto injector. The separation of EANT was executed by a Phenomenex Luna 5 µ C18(2) 100 Å column (250 × 4.6 mm, flow rate = 1.0 mL/min) eluted with two solvents [solvent A = 0.1% trifluoroacetic acid in aqueous solution; solvent B = acetonitrile]. The gradient program was set up as (a) 0 min (20% B), (b) 60 min (100% B), (c) 70 min (100% B), (d) 80 min (20% B). The fingerprint profile and separation method of EANT are shown in Supplementary Figure S1 and Table S1, respectively.

The plant material of *N. thorellii* x (*ventricosa* x *maxima*) was collected from KBCC. However, it is hard to obtain large amounts of this plant for active component separation and identification. Therefore, we bought another Nepenthes plant (*N. mirabilis*) from a local drug store in China. Because taxonomically related plants usually produce similar secondary metabolites (chemotaxonomic approaches), we used the major compounds isolated from *N. mirabilis* as standard to interpret the components in the fingerprint profile of our research material. We also used repeated column chromatography to isolate those major compounds from *N. mirabilis*. Those major compounds were identified by their NMR and mass spectroscopic data.

### 4.3. Cell Culture and Viability

Human breast cancer cells (MCF7 and SKBR3) were obtained from the American Type Culture Collection (ATCC, Manassas, VA, USA) and cultured in Dulbecco’s Modified Eagle’s Medium (DMEM) (Gibco, Grand Island, NY, USA). Human breast normal cells (M10) were obtained from Bioresource Collection and Research Center (BCRC) (Hsinchu, Taiwan) and cultured in Minimum Essential Medium Alpha Medium (αMEM) (Gibco). All cells were supplemented with 10% fetal bovine serum (Gibco) and antibiotics, and maintained in a humidified 5% CO2 incubator at 37 °C. Cell viability was determined by Promega MTS Assay (Madison, WI, USA) as previously described [27].

### 4.4. Cell Cycle Assay

Cell cycle distribution was determined using 1 μg/mL of 7-aminoactinmycin D (7AAD) (Biotium, Inc., Hayward, CA, USA) for 30 min [40]. Following fixation and washing, cells were suspended in PBS for flow cytometry (Accuri C6, Becton-Dickinson, Mansfield, MA, USA).

### 4.5. Annexin V/7AAD Assay for Apoptosis

Cells were incubated with annexin V/7AAD reagents (Strong Biotech Corporation, Taipei, Taiwan) for 30 min [7]. Following suspension in PBS, apoptotic cells were counted by flow cytometry (Accuri C6).

### 4.6. ROS Assay

Cells were incubated with 2 μM of 2′,7′-dichlorodihydrofluorescein diacetate (DCFH-DA) (Sigma-Aldrich, St. Louis, MO, USA) for 30 min at 37 °C [41]. Following suspension in PBS, intracellular ROS levels were measured by flow cytometry (Accuri C6).

### 4.7. GSH Assay

Cells were incubated with 0.1 μM of CellTracker™Green CMFDA (5-chloromethylfluorescein) (Thermo Fisher Scientific, Carlsbad, CA, USA) for 30 min at 37 °C [42]. Following suspension in PBS, intracellular ROS levels were measured by flow cytometry (Accuri C6).

### 4.8. Mitochondrial Superoxide Assay

Cells were incubated with 5 μM of MitoSOX™ Red (Molecular Probes, Invitrogen, Eugene, OR, USA) for 30 min at 37 °C [43]. Following suspension in PBS, MitoSOX levels were measured by flow cytometry (Accuri C6).

### 4.9. Mitochondrial Membrane Potential Assay

Cells were incubated with 50 nM of DiOC_2_(3) from the MitoProbe^TM^ kit (Invitrogen, San Diego, CA, USA) for 20 min [44]. Following suspension in PBS, MMP levels were measured by flow cytometry (Accuri C6).

### 4.10. γH2AX Assay

Following fixation, cells were incubated with the primary antibody (1:50 dilution) against p-Histone H2A.X (γH2AX) (Santa Cruz Biotechnology, Santa Cruz, CA, USA) for 1 h at 4 °C [45]. Subsequently, cells were incubated with Alexa Fluor^®^488-conjugated secondary antibody (Cell Signaling Technology) (1:10,000 dilution) for 30 min at room temperature. Finally, cells were stained with 1 μg/mL of 7AAD for 30 min. Following suspension in PBS, γH2AX levels were measured by flow cytometry (Accuri C6).

### 4.11. 8-Oxo-2′deoxyguanosine Assay

Following fixation, cells were incubated with the FITC-conjugated antibody (1:100 dilution) against 8-OxodG (Santa Cruz Biotechnology) for 1 h. Following suspension in PBS, 8-OxodG levels were measured by flow cytometry (Accuri C6).

### 4.12. Statistical Analysis

Data were analyzed by JMP12 software (SAS Institute, Cary, NC, USA). The significance between multiple comparisons was analyzed by one-way analysis of variance (ANOVA) using the Tukey HSD post-hoc test. Different treatments with the same small letters indicate non-significance, while different treatments without the same small letters indicate significance.

## Supplementary Materials

Supplementary materials can be found at https://www.mdpi.com/1422-0067/20/13/3238/s1. Supplementary Table S1. The HPLC method for fingerprint profile of *N. thorellii* x (*ventricosa* x *maxima*). Supplementary Figure S1. Components of EANT. (A) Fingerprint profile of EANT. It is monitored at 365 nm. (B) Retention time of (NT-B). Volume is 10 μL. It is monitored at 254 nm. (D) Retention time of quercetin 3-*O*-(6″-*n*-butyl β-D-glucuronide) (NT-E). Volume is 10 μL. It is monitored at 254 nm. UV patterns were inserted in right side for the Supplementary Figure S1B–D. Supplementary Figure S2. Patterns and statistics for positive controls for flow cytometry experiments using breast cancer SKBR3 and MCF7 cells. (A, B) Cell cycle, (C, D) apoptosis, (E, F) ROS, (G, H) GSH, (I, J) MitoSOX, (K, L) MMP, (M, N) γH2AX, and (O, P) 8-oxodG.
