# Correction: Ou-Yang, F. et al. Antiproliferation for Breast Cancer Cells by Ethyl Acetate Extract of *Nepenthes thorellii* x (*ventricosa* x *maxima*). *Int. J. Mol. Sci.* 2019, *20*, 3238

**DOI:** 10.3390/ijms22020668

**Published:** 2021-01-12

**Authors:** Fu Ou-Yang, I-Hsuan Tsai, Jen-Yang Tang, Ching-Yu Yen, Yuan-Bin Cheng, Ammad Ahmad Farooqi, Shu-Rong Chen, Szu-Yin Yu, Jun-Kai Kao, Hsueh-Wei Chang

**Affiliations:** 1 Division of Breast Surgery and Department of Surgery, Kaohsiung Medical University Hospital, Kaohsiung 80708, Taiwan; kmufrank@gmail.com; 2 Cancer Center, Kaohsiung Medical University Hospital, Kaohsiung 80708, Taiwan; 3 Department of Biomedical Science and Environmental Biology, Kaohsiung Medical University, Kaohsiung 80708, Taiwan; s0932961465@gmail.com; 4 Department of Radiation Oncology, Faculty of Medicine, College of Medicine, Kaohsiung Medical University, Kaohsiung 80708, Taiwan; reyata@kmu.edu.tw; 5 Department of Radiation Oncology, Kaohsiung Medical University Hospital, Kaohsiung 80708, Taiwan; 6 Department of Oral and Maxillofacial Surgery Chi-Mei Medical Center, Tainan 71004, Taiwan; ycysmc@gmail.com; 7 School of Dentistry, Taipei Medical University, Taipei 11050, Taiwan; 8 Graduate Institute of Natural Products, Kaohsiung Medical University, Kaohsiung 80708, Taiwan; jmb@kmu.edu.tw (Y.-B.C.); highshorter@hotmail.com (S.-R.C.); s91412232@gmail.com (S.-Y.Y.); 9 Institute of Biomedical and Genetic Engineering (IBGE), Islamabad 44000, Pakistan; ammadfarooqi@rlmclahore.com; 10 Institute of Biomedical Sciences, National Chung Hsing University, Taichung 40227, Taiwan; 11 Pediatric Department, Children’s Hospital, Changhua Christian Hospital, Changhua 50006, Taiwan; 12 School of Medicine, Kaohsiung Medical University, Kaohsiung 80708, Taiwan; 13 Drug Development and Value Creation Research Center, Kaohsiung Medical University, Kaohsiung 80708, Taiwan; 14 Institute of Medical Science and Technology, National Sun Yat-sen University, Kaohsiung 80424, Taiwan; 15 Department of Medical Research, Kaohsiung Medical University Hospital, Kaohsiung 80708, Taiwan

The authors would like to make corrections to their published paper [1].

There were mistakes in some usages of the chemical name “isoplumbagin” in the original version in Sections 2.1, 3.1, and 3.2. These “isoplumbagin” words should be changed to “plumbagin”.

Literature reported that the difference between plumbagin and isoplumbagin is the coupling constant of methyl group (1.5 Hz for plumbagin and 1.2 Hz for isoplumbagin) [2]. We found that the ^1^H and ^13^C spectra data (Figure 1 and Figure 2) of the main compound of EANT is plumbagin because our compound shows a *J* value of 1.5. Therefore, we confirmed the major compound of EANT to be “plumbagin” based on spectra data rather than isoplumbagin.

We also further performed the detailed physical characters such as melting temperature. The melting points of these two compounds are different (74–75 °C for plumbagin and 158–159 °C for isoplumbagin) [2]. After checking the melting point (77–78 °C) of the major compound isolated from EANT, we realized it should be plumbagin rather than isoplumbagin. 

Additionally, the paragraph for the Supplementary Materials also needs to be corrected due to missing words. The authors have corrected the error as shown below. The change does not affect the scientific results. The authors would like to apologize for any inconvenience that may have been caused to readers of the journal. The manuscript will be updated, and the original will remain online on the article webpage.

Please find the correct sentences below (only isoplumbagin in the original paper has been corrected to plumbagin):

2.1. The Identified Components from Fingerprint Profiles of EANT (Page 2: Line 2 of the First Paragraph)

According to HPLC fingerprinting assay (Supplementary Figure S1), the major bioactive components of EANT are plumbagin, *cis*-isoshinanolone, quercetin 3-*O*-(6″-*n*-butyl β-d-glucuronide), and fatty acids.

3.1. EANT Preferentially Inhibits Proliferation of Breast Cancer Cells (page 8: line 6 of the Second Paragraph of 3.1)

In the current study, we found that the major bioactive components of EANT identified by HPLC fingerprinting method were plumbagin [21], *cis*-isoshinanolone [22], and quercetin 3-*O*-(6″-*n*-butyl β-d-glucuronide) [23] (Supplementary Figure S1).

3.2. EANT Induces Oxidative Stress on Breast Cancer Cells (Page 8: line 1 of the Third Paragraph of 3.2)

Plumbagin is a common naphthoquinone in Nepenthes. Moreover, plumbagin but not isoplumbagin is identified in EANT.

Supplementary Materials (page 11)

The following are available online at http://www.mdpi.com/1422-0067/20/13/3238/s1, Table S1: The HPLC method for fingerprint profile of *N. thorellii* x (*ventricosa* x *maxima*), Figure S1: Components of EANT. (A) Fingerprint profile of EANT. It is monitored at 365 nm. (B) Retention time of plumbagin (NT-A). Volume is 50 μL. It is monitored at 400 nm. (C) Retention time of *cis*-isoshinanolone (NT-B). Volume is 10 μL. It is monitored at 254 nm.

## Figures and Tables

**Figure 1:**
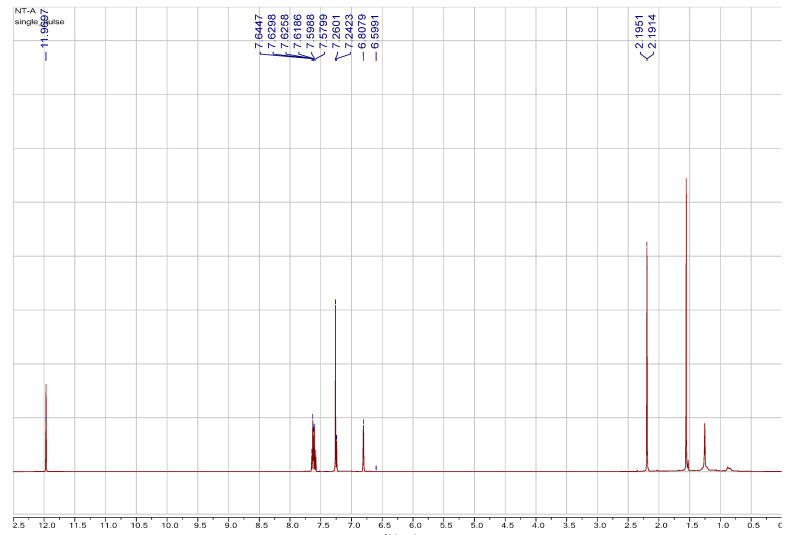
Spectrum data of ^1^H of the major compound isolated from EANT.

**Figure 2:**
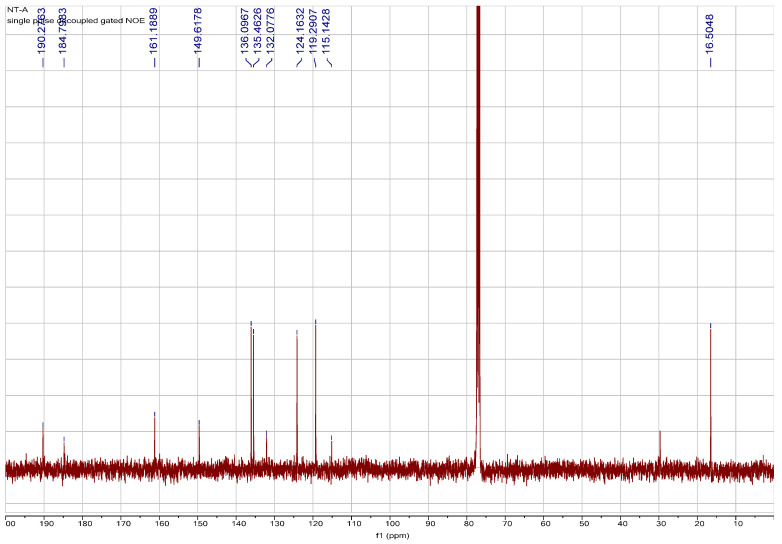
Spectrum data of ^13^C of the major compound isolated from EANT.
