# Supplementary material for: Antiproliferation for Breast Cancer Cells by Ethyl Acetate Extract of Nepenthes thorellii x (ventricosa x maxima)
Source: Int J Mol Sci. 2019 Jul 1;20(13):3238. doi: 10.3390/ijms20133238 (PMC6651324; doi:10.3390/ijms20133238)

Supplementary Figure 1. Components of EANT. (A) Fingerprint profile of EANT. It is monitored at 365 nm. (B) Retention time of isoplumbagin (NT-A). Volume is 50 µL. It is monitored at 400 nm. (C) Retention time of *cis*-isoshinanolone (NT-B). Volume is 10 µL. It is monitored at 254 nm. (D) Retention time of quercetin 3-O-(6''-n-butyl β-D-glucuronide) (NT-E). Volume is 10 µL. It is monitored at 254 nm. UV patterns were inserted in right side for the Supplementary Figures 1B, 1C and 1D.

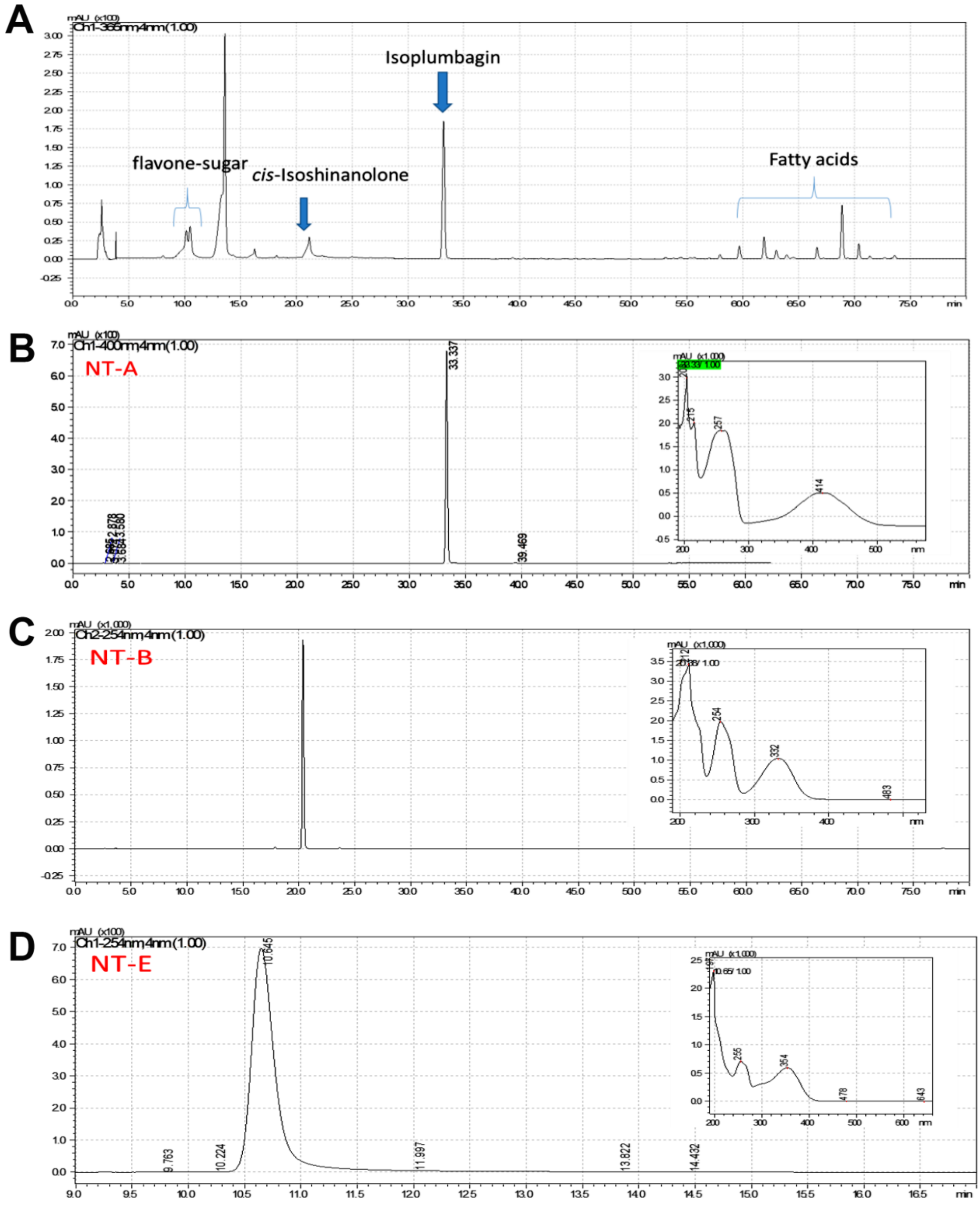

Supplement: Supplementary file 1 [file ijms-20-03238-s001.zip › Supplementary Figure 1.pdf]
