# Supplementary material for: Antiproliferation for Breast Cancer Cells by Ethyl Acetate Extract of Nepenthes thorellii x (ventricosa x maxima)
Source: Int J Mol Sci. 2019 Jul 1;20(13):3238. doi: 10.3390/ijms20133238 (PMC6651324; doi:10.3390/ijms20133238)
Supplement: Supplementary file 1 [file ijms-20-03238-s001.zip › Supplementary Table 1.pdf]

Supplementary Table 1. The HPLC method for fingerprint profile of *Nepenthes thorellii* x (*ventricosa* x *maxima*).

<table>
<tr><td>HPLC</td><td>Shimadzu SIL-10AD VP auto injector
Shimadzu LC-20AD prominence liquid chromatograph
Shimadzu SPD-M10A VP diode array detector</td></tr>
<tr><td>Solid phase</td><td>Phenomenex Luna 5 μ C18(2) 100A
250*4.60 mm, 5 μ
P/NO 00G-4252-E0</td></tr>
<tr><td>Mobile phase</td><td>A: 0.1% $TFA_{(aq)}$
B: ACN
<table>
<tr><th></th><th>Time</th><th>Module</th><th>Action</th><th>Value</th></tr>
<tr><td>1</td><td>0.10</td><td>Pumps</td><td>B.Conc</td><td>20</td></tr>
<tr><td>2</td><td>60.00</td><td>Pumps</td><td>B.Conc</td><td>100</td></tr>
<tr><td>3</td><td>70.00</td><td>Pumps</td><td>B.Conc</td><td>100</td></tr>
<tr><td>4</td><td>80.00</td><td>Pumps</td><td>B.Conc</td><td>20</td></tr>
<tr><td>5</td><td>90.00</td><td>Pumps</td><td>B.Conc</td><td>20</td></tr>
<tr><td>6</td><td>90.00</td><td>Controller</td><td>Stop</td><td></td></tr>
</table></td></tr>
<tr><td>Flow rate</td><td>1 mL/min</td></tr>
<tr><td>Analysis time</td><td>60 mins</td></tr>
<tr><td>Pressure</td><td>112 kgf (20% $ACN_{(aq)}$)</td></tr>
<tr><td>Injection volume</td><td>50 μL</td></tr>
<tr><td>Sample</td><td>EANT (1 mg/mL, in MeOH)
Isoplumbagin (1 mg/mL, in MeOH)
cis-Isoshinanolone (1 mg/mL, in MeOH)
Quercetin 3-O-(6"-n-butyl β-D-glucuronide) (1 mg/mL, in MeOH)</td></tr>
<tr><td>Wavelength</td><td>210 nm, 254 nm</td></tr>
</table>
